# Teaching quantitative ecology online: An evidence‐based prescription of best practices

**DOI:** 10.1002/ece3.6607

**Published:** 2020-09-02

**Authors:** Miguel A. Acevedo

**Affiliations:** ^1^ Department of Wildlife Ecology and Conservation University of Florida Gainesville Florida USA

**Keywords:** active learning, ecological modeling, online learning, pedagogy, quantitative ecology, statistics

## Abstract

Quantitative skills are becoming central to the undergraduate and graduate curriculum in ecology and evolutionary biology. While previous studies acknowledge that students perceive their quantitative training to be inadequate, there is little guidance on best practices. Moreover, with the recent COVID‐19 sudden transition to online learning, there is even less guidance on how to effectively teach quantitative ecology online. Here, I synthesize a prescription of pedagogical best practices for teaching quantitative ecology online based on a broad review of the literature on multiple quantitative disciplines. These best practices include the following: (1) design and implement the class to meet learning goals using online strategies specifically; (2) create an open, inclusive, and welcoming online environment that promotes a sense of learning community; (3) acknowledge the diversity of talents and learning strategies; (4) use real‐world examples and assessments; (5) account for gaps in knowledge; (6) emphasize the modeling cycle process; (7) focus on developing ideas rather than tools or procedures; (8) if needed, introduce computational tools thoroughly before combining them with mathematical or statistical concepts; (9) evaluate the course constantly; and (10) put your heart and soul into the class. I hope these practices help fellow instructors of quantitative ecology facing similar challenges in providing our students with the knowledge and skills needed to meet the challenges of the future.

## Graphical abstract

Quantitative skills are becoming central to the undergraduate and graduate curriculum in ecology and evolutionary biology. While previous studies acknowledge that students perceive their quantitative training to be inadequate, there is little guidance on best practices. I synthesized a prescription of pedagogical best practices for teaching quantitative ecology online based on a broad review of the literature on multiple quantitative disciplines.

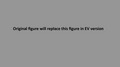

## 1 INTRODUCTION

Ecology is becoming an increasingly quantitative discipline. In 2012, more than 70% of studies published in popular ecology journals applied some type of advanced statistical model (Barraquand et al., 2014). Still, most students feel their quantitative skills are inadequate and there is a recent emphasis on incorporating more quantitative content into undergraduate and graduate courses in ecology and evolutionary biology (EEB) (e.g., Chen, Scott, & Stevens, 2018; Colon‐Berlingeri & Burrowes, 2011; Guzman, Pennell, Nikelski, & Srivastava, 2019; Rahn, Willner, Deverick, Kemper, & Saha, 2019). Teaching quantitative skills to nonmath majors is challenging, and many instructors prefer to teach in classroom environment because it provides more support for lower performing students than online settings (Lu & Lemonde, 2013). However, due to the COVID‐19 pandemic, many of us had to suddenly transition our courses online without necessarily having the training to design the course appropriately.

To address this issue, I conducted a literature review to identify evidence‐based best practices to teach quantitative courses online. In this review, I considered studies on asynchronous and synchronous online delivery for undergraduate and graduate students, but not hybrid or flipped methods. Also, for the purpose of this review, I broadly define quantitative ecology as those courses that have a prevalent component of mathematics or statistics. These can include population ecology, statistical ecology, theoretical ecology, community ecology, landscape ecology, and wildlife management among others. The pedagogical literature specific for quantitative ecology is limited. Therefore, I complemented the review with literature of pedagogical research in related disciplines such as mathematics, statistics, economics, engineering, and public health. Below, I synthesize what I learned as a prescriptive list of best practices. I acknowledge this topic deserves a longer and thorough discussion than a prescriptive list. Yet, I chose this format to address the immediate need to have a quick guide for instructors teaching quantitative ecology online for the first time.

An effective pedagogy of quantitative ecology online must address common challenges in the pedagogy of mathematics such as math anxiety (Betz, 1978; Foley et al., 2017). This type of anxiety decreases student's performance by disrupting cognitive processing and working memory (Ashcraft, 2002). Intergenerational transmission of low math achievements and math anxiety can be the result of a genetic component or learned behavior (Maloney, Ramirez, Gunderson, Levine, & Beilock, 2015; Wang et al., 2014). The student's past negative experiences in school can also shape their perceptions and anxiety in college many times driven by their interactions with their teachers (O'Leary, Fitzpatrick, & Hallett, 2017). In addition, the instructor's anxiety has a significant negative effect on the student's achievements. The root of the relationship between math anxiety and performance is thought to be that worrying replaces the thinking and reasoning resources needed to perform mathematical tasks (Beilock & Maloney, 2015). While remediation of math anxiety goes beyond the purpose of this review, math anxiety is such a prevalent issue that any quantitative ecology course serves students best if the issue is addressed (see below).

Does online teaching of quantitative ecology increase math anxiety compared to a classroom setting? Early studies showed that 25%–50% of adults, including first‐year undergraduate students, had anxiety related to using computers and technology (Rosen & Maguire, 1990; Weinberg & Fuerst, 1984). While this percentage is expected to decrease with time as technology becomes more prevalent in education, there is still prevalent anxiety in using computer and technology as educational resources (Maymon, Hall, & Goetz, 2018). Therefore, teaching in an online environment may amplify some of these anxious feelings in students. There is an interesting case study by DeVaney (2010) where he compared anxiety and general attitudes toward statistics in a graduate class in online versus face‐to‐face classes. He found that online students had higher levels of anxiety and negative attitudes toward statistics than students on campus. Yet, these negative attitudes improved throughout the 10‐week course, which offers encouragement that by applying the right resources, we can transform these attitudes and improve learning outcomes. The list of best practices below is intended to provide guidance in this process.

Below, I enumerate a list of 10 best practices for teaching quantitative ecology online. Most of these suggestions are rooted on constructivist pedagogy under an active learning paradigm. I consider computational and quantitative skills to be complementary but separate (see item number 8). Therefore, this list of best practices emphasizes the quantitative/modeling aspect of the online course. For best practices for teaching computational skills, I encourage the reader to look at the extensive literature in the pedagogy of computer science (e.g., Wade, 2018).

## 2 BEST PRACTICES

1. Design and implement the class to meet learning goals using online strategies specifically. During the COVID‐19 transition to an online format, many of us were asked to “move” or “adapt” our courses to an online format. This transition was necessary given the circumstances to protect the life and health of our faculty and students. In an ideal situation, we want to design our course to meet the expected learning outcomes with pedagogical techniques specific to online teaching (Fish & Wickersham, 2009). To this end, we can apply principles of backward design and student journey mapping. Backward design emphasizes identifying specific learning goals at the beginning of the design process (Wiggins & McTighe, 1998). Then, we design a set of lessons, exercises, and assessments that will best fulfill these learning outcomes specifically in an online setting. This design strategy allows us to focus on learning outcomes instead of simply delivering content. Student journey mapping is a complementary strategy that, borrowing concepts from design thinking, encourages instructors to think on the class from a student perspective (Andrews & Eade, 2013). This design process should also include strategies for seamless implementation. Students thrive in a structured and well‐organized class with clearly defined learning outcomes, activities, and assessment (Ben‐Zvi, Gravemeijer, & Ainley, 2018). The starting point should be a well‐organized and visually welcoming learning managing system such as canvas. This can be combined with a GitHub page (or any other similar platform such as Open Science, Bitbucket, or SourceForge) that stores data and for use in computational exercises. Following the proposed, schedule with minimal deviations helps maintain structure. Also, having the same weekly due dates (e.g., exercises are due every week on Wednesday before 5 p.m.) helps students plan better and accommodate their multiple responsibilities. Lastly, using the right technology to meet learning outcomes is key. Overall, we should use technology to make the class more active and engaging. In online classes, students prefer courses in which complex topics are split into manageable smaller modules. These modules can include short videos (<10 min) that present the instructor alongside the slides (Meseguer‐Martinez, Ros‐Galvez, & Rosa‐Garcia, 2017). These slides should be simple with each making 1–3 points (Garrett, 2016). A formative assessment or low‐stake quiz after each lecture helps maintain student engagement (Dobson, 2008). Online group discussions can help maintain engagement, but also a sense of learning community that improves students' performance (see below; Dalelio, 2013).
2. Create an open, inclusive, and welcoming online environment that promotes a sense of learning community. Student support and continuous communication, with the instructor and among students, is the cornerstone to create a sense of learning community. With high school students, the support of family members is key to ameliorate their math anxieties (Finlayson, 2014). This support group may lack for undergraduate and graduate students, but it can be replaced by an extended community of classmates and supporting instructors in an engaged online community. Here, I list a few ideas to build a supportive learning community. (a) Foster a learning community where students feel free to express their views and make mistakes in a safe environment (Teal et al., 2015, e.g., Data Carpentry's code of conduct). (b) Provide opportunities for interactions. Group learning has vast empirical support showing that it improves performance in quantitative courses (Charalambous, Hodge, & Ippolito, 2020; Paterson & Sneddon, 2011; Springer, Stanne, & Donovan, 1999). In online settings, small group discussions and “think, pair, share” activities can be conducted using breakout rooms in Zoom or similar features in other online meeting applications. Discussion boards have mixed support in the literature (Thomas, 2002); however, if well implemented, they can help develop skills at top levels of Bloom's taxonomy (Bachner & O’Byrne, 2019). Briefly, Bloom's taxonomy is a classification of learning objectives based on level of skill varying from remembering to creating (Anderson, Bloom, & Kathwohl, 2001). For example, an instructor can pose a problem in the discussion board and encourage students describe *how* they solve it. (c) Feedback should be quick, personalized, and constructive. This constructive feedback addresses the learner by name, is balanced, specific, uses a positive tone, and asks questions promoting thinking (Leibold & Schwarz, 2015). Online courses are a great place to apply video feedback—where the instructor records a short video providing comments or suggestions on assessments or assignments. Students prefer video feedback to traditional feedback in text because it is more personal (Robinson, Centifanti, Brewer, & Holyoak, 2015). Instructors may also find video feedback time‐efficient because the time required is similar to the feedback in text, but the benefits are greater. Large‐enrollment online classes may require the use of multiple instructors and/or teaching assistants to provide timely feedback (Stefan, Gutlerner, Born, & Springer, 2015). (d) Use humor to relieve technological and math anxieties. Forte (1995) in his article titled “Teaching statistics without the sadistics” argues for the use of play and humor as an evidence‐based technique to reduce math anxiety and improve students' performance in statistics courses (Schacht & Stewart, 1990). Math or statistics jokes can help promote a lighter tone in the class and keep students interested. For example, in synchronous online classes we can post a clever math joke in the waiting room right before starting class while the students are getting ready to start. If humor is not your strength, just keeping a positive and engaging attitude, praising students when they succeed, and reminding them of their progress may go a long way to foster a welcoming environment. There are other evidence‐based techniques that we can teach our students to address math anxiety. For instance, background instrumental music, focus breathing exercises, cooperative learning exercises, expressive writing, and other reappraisal techniques have been shown to ameliorate these anxious feelings (Brunyé et al., 2013; Feng, Suri, & Bell, 2014; Lavasani & Khandan, 2011; Park, Ramirez, & Beilock, 2014).
3. Acknowledgement the diversity of talents and learning strategies. We have been more mindful of the diversity in our skills and abilities since Howard Gardner popularized his multiple intelligence ideas in the 80s (Barrington, 2004; Gardner, 1992). Still, we often target our teaching toward the “average” student in a class using a single pedagogical strategy resulting in an unintentional bias against underprivileged students (Nelson, 1996). A few simple changes may help ameliorate this issue. Repetition has been a persistent principle in educational research. New research is showing that learning can be enhanced by repetition with increased sensorimotor variability (Wymbs, Bastian, & Celnik, 2016). This means that teaching the material multiple times but in different ways may improve students' learning than simple repetition. For instance, to teach simple linear models we can start with a synchronous lecture or a set of asynchronous videos discussing the theoretical principles and equations. These lectures can be complemented by discussing a peer‐reviewed paper that applies linear models to a real‐world problem. We can also use the data from the paper to replicate the analyses in groups using R or python. This computational exercise will allow the students to learn how the equations are translated into code. By combining all three methods (theory, real‐world example, and replicate analysis), students may learn similar principles while experiencing sensorimotor variability. This method also benefits from leveraging multiple learning abilities.
4. Use real‐world examples and assessments. Making the connection between mathematical models and real‐world examples is the most common challenge faced by students in applied mathematics courses (Chang, 2011). An abstract style of teaching based only on proofs and algebraic demonstrations is inadequate to keep students interested and motivated (Abramovich & Grinshpan, 2008). Instead, we can improve student's motivation by incorporating real‐world examples into class discussions and assessments (Abdulwahed, Jaworski, & Crawford, 2012). There are multiple data repositories such as Dryad, Figshare, or Open Science Framework that store data sets from peer‐reviewed papers that can be used as case studies in class. By re‐analyzing data from published papers, we are also encouraging practices of open science and reproducibility. We can also use these real‐world examples strategically to promote diversity. For instance, real‐world examples from multiple biomes and geographic regions encourage a global perspective. Similarly, including case studies from authors belonging to underrepresented minorities can promote inclusion and a sense of belonging to minority students that are severely underrepresented in EEB (Armstrong, Berkowitz, Dyer, & Taylor, 2007; O'Brien, Bart, & Garcia, 2020). While using real‐world examples strategically can help students’ motivation and promote diversity and inclusion, there is also value in the use of simulated data to teach the interaction between the model and the data generation process (Tintle et al., 2015). Simulated data are simple and clean allowing students to focus on the mathematical concepts instead of data noise. While the pedagogy literature argues for a “varied diet” of assessments that combine different types of formative and summative assessments, closed‐book summative assessments are the norm in undergraduate quantitative courses (Iannone & Simpson, 2011). Yet, students generally underperform in high‐stake assessments due to increased anxiety (Agboola & Hiatt, 2017; Iannone & Simpson, 2011). Instead, homework problems are the assessment that students find has the greatest impact on their learning (Chow, 2015; Glass & Sue, 2008). Solving problems individually or in groups mirrors better what quantitative ecologists do professionally. Lastly, in online settings, it is key that the instructions for these assessments are very clear. This is particularly true for asynchronous classes where students cannot ask for clarifications in real time.
5. Account for gaps in knowledge. Students come to our classrooms with a wide variety of mathematical backgrounds. Some have taken advanced calculus classes in high school and continued to take other mathematics and statistics courses as part of their undergraduate degree. Others may have a limited background in mathematics and even chosen a career in EEB to avoid quantitative topics. When these students enroll in our courses, we make assumptions about what quantitative skills students have. First, I suggest being explicit about these assumed skills in the syllabus. Stating per‐required courses helps, but it is more helpful to state what specific topics or skills you are assuming students have and at what level of mastery. For example, I assume that students took Calculus I and that they remember *qualitatively* that you can use calculus to find the maximum or minimum points of a function. This knowledge is important when we discuss maximum likelihood estimation. However, I do not assume that they remember every single differentiation rule. Remembering the rules in detail is less relevant in my courses because we often use software to calculate maximum likelihoods. Second, we have to meet students where they are and bridge the gap in knowledge (Abdulwahed et al., 2012; King & Cattlin, 2015). Yes, this means that you will likely have to reteach a few mathematical skills that students “should have” learned before. But the alternative is to assume they remember everything or that they will go and review on their own time which is unrealistic. There are multiple options to deliver this review content. Some use lessons available online on YouTube or Kahn Academy. Alternatively, we can develop our online lessons where we teach the content that is specifically needed for our courses. This latter strategy may be more time‐consuming at first, but it provides the students with exactly the skills they need to meet the learning goals of our courses.
6. Emphasize the modeling cycle process. The modeling cycle summarizes the systematic process of describing a system using mathematical models. It is the core concept of any quantitative ecology class. This model building process can be described as a cycle that varies depending on the type of model or application (e.g., Bolker, 2008; Galbraith & Stillman, 2006). It can be generally summarized as follows (adapted from Geiger et al., 2018): (a) identify a real‐life problem (question or hypothesis); (b) identify the key components of the problem and simplify it to develop a workable model while being explicit about assumptions and missing information; (c) transform it into a schematic figure and then into an idealized mathematical model (i.e., “mathematizing”); (d) generate an initial solution; (e) contrast the initial solution with the original problem and consider its validity; and (f) revise until a valid result is attained. To teach this concept at the basic level of Bloom's taxonomy, we can assign a modeling paper and ask students to identify the components of the modeling cycle. To promote higher levels of understanding, we can ask students to develop a modeling cycle for a real‐world example. In an online setting, it will be useful to break students into small groups to work on these issues and then report back to their process in a short video. Each video can have a discussion board where other students can ask questions and share ideas. It is important to emphasize to the students that applying the modeling cycle to ecological problems is challenging and the procedure is not as linear as it seems. It is good practice to identify common issues such as meeting assumptions, missing data, and identifiability issues, and discuss general ways to overcome them.
7. Focus on developing ideas rather than tools or procedures. While there is pedagogical merit in students’ learning and repeating procedures, ideally, we want students to also learn at higher levels of Bloom's taxonomy including developing and evaluating models (Ben‐Zvi et al., 2018). Teaching procedures emphasize the rules used to solve a problem in a descriptive way without building connected meaning to support them (Hiebert, 2013). An example from quantitative wildlife ecology can include teaching how to use the Lincoln–Petersen equation to estimate animal abundance without discussing its roots in the idea of known ratios. In contrast, we can teach the concept of known ratios first and derive the Lincoln–Petersen estimation from it. By teaching the concepts, first we give the students the skills to apply knowledge to solve new problems. In other words, procedural knowledge is useful but has limited problem‐solving applications, while conceptual knowledge is more flexible and can lead to innovation (Engelbrecht, Harding, & Potgieter, 2005). As technology improves, many of the procedures used in quantitative ecology may change. Think about how ecological statistics have changed in the last 20 years! However, statistical concepts such as distributions, uncertainty, and inference remain. Therefore, building a strong conceptual toolkit in our students will better prepare them to face future challenges.
8. If needed, introduce computational tools thoroughly before combining them with mathematical or statistical concepts. Both computational and quantitative tools are fundamental in all areas of ecology from theoretical to applied. Computational exercises can be very useful tools to teach complex quantitative topics. Still, recent research emphasizes that modeling and programming are fundamentally different skills (Auker & Barthelmess, 2020). For many students, our quantitative ecology courses would be their first experience with computer programming. Teaching skills simultaneously hinder learning and amplify anxiety (Sana, Weston, & Cepeda, 2013). To prevent this, it would be ideal if students take an introductory class on data management and visualization in R or python as a prerequirement of a quantitative ecology class. In cases where this is not possible, it would be ideal to have a thorough introduction to programming thinking at the beginning of class before integrating ecological modeling concepts. Addressing students' issues with their hardware is challenging in online courses because physical access to the students' computers is restricted. To ameliorate this issue, we can be very specific about hardware recommendations. For most courses, an ideal hardware setting will include a recent computer (<3 years old) running Windows, macOS, or GNU/Linux with enough capacity and memory to install new software. An instructor can make instructional videos or screencasts explaining how to properly download and install the software in different OS. Chromebooks are popular among students because they are light and affordable, but currently, it is difficult to install statistical software in them. Alternatively, an instructor can provide access to a server that has access to the software such as RStudio server. This service provides a browser‐based interface with all necessary packages pre‐installed that can be accessed using any type of OS including Chromebooks or tables. Still, server‐based access has the downside that the student needs to have a reliable Internet connection, which can be challenging to some if they are working from their homes.
9. Evaluate the course constantly. We should adaptively improve our courses constantly based on student's feedback. Although the usefulness of the end of the semester student evaluations has been debated (Wachtel, 1998), they are useful as part of a holistic process of evaluation. But if the students need to wait until the end of the semester to provide their feedback, it results in little to no benefit to them. Mid‐semester evaluations are useful to address this issue. They have many forms, and many institutions have University‐wide efforts in place. For many years, I had a simple online form with three blanks to fill: continue, start, and stop. Students anonymously can let me know which teaching strategies they find useful and that I should continue to use, teaching strategies from other instructors that they suggest I start using, and activities that I should stop doing because they provide little benefit to their learning. In addition to end and mid‐semester evaluations, instructors should ask for informal feedback constantly from students. If teaching synchronously, instructors can use the waiting room to ask the students their perspectives on class activities. If teaching asynchronously, each class activity can include an anonymous query where students can share their perspectives.
10. Put your heart and soul into the class. Being enthusiastic about the topic is an easy way to improve performance because the instructor's attitudes get transmitted to the students (Keller, Hoy, Goetz, & Frenzel, 2016). This enthusiasm can derive from our passion for teaching or our excitement about quantitative ecology. Enthusiasm and motivation can also stem from recognizing that we have an enormous responsibility to train our students for the challenges they will face in their professional lives. Many of our students will go to work in academia, but others will go to work in NGOs, or state or federal agencies and will be in charge of managing the future of our natural resources. The amount of effort and passion that we put into our courses will pay dividends as our students face the challenges of the future with solid quantitative training.

## 3 DISCUSSION

This nonexhaustive list of best practices to teach quantitative ecology online needs to be contextualized in students' and instructors' perspectives on online teaching. How does student performance compare between online and in classroom formats? Empirical studies show much variability. For example, in a univariate statistics graduate course, students generally performed 84% better in a face‐to‐face setting compared to online (Caviglia‐Harris, 2016). However, females performed better online, while males performed better in the physical classroom. In another study, in the topic of derivation (Calculus I), online students had 5% better achievement (Gürsul & Keser, 2009). Interestingly, a study on an undergraduate health sciences statistics class shows that academically performing students had comparable test results between face‐to‐face and online settings. In contrast, underperforming students had lower test results in the online setting (Lu & Lemonde, 2013). These studies show that a student's performance is context‐dependent. This context includes the idiosyncrasies of each class in combination with student's traits and characteristics.

Students' values toward math influence their academic achievement and behavioral outcomes (Andrews, Runyon, & Aikens, 2017). For many students, adapting to the new experience of taking classes online is a real challenge (Fish & Wickersham, 2009). How can we help students overcome this challenge? The pedagogical literature on online teaching suggests promoting complementary skills such as time management, metacognition, effort regulation, and critical thinking (Broadbent & Poon, 2015). Also, promoting a positive attitude and digital literacy increases self‐efficacy, a key characteristic of students that perform well in quantitative courses (Prior, Mazanov, Meacheam, Heaslip, & Hanson, 2016). Therefore, the key for student's achievement in online quantitative ecology courses goes beyond designing activities that meet our specific learning outcomes but also help students develop a complementary set of skills that help them adapt to the online delivery system. These additional skills will also help them in their transition to the workforce.

While understanding students' perspectives is key to effectively design an online class in quantitative ecology, we also need to acknowledge that instructors' perspectives also matter. Many instructors believe that online teaching is more time‐demanding (Bolliger & Wasilik, 2009). A study found that online teaching takes a minimum of 14% more time than face‐to‐face. However, other studies argue that time per‐student is similar to face‐to‐face delivery (Hislop & Ellis, 2004), while others found that online teaching takes, on average 11.28 min less per student than face‐to‐face classes (Van de Vord & Pogue, 2012). Every class is different, and this variability likely stems from the subtle differences between courses. What is clear is that designing an effective online course in quantitative ecology takes time, effort, and dedication. For instructors that are thinking of going back to face‐to‐face instruction after teaching online, there is evidence showing that teaching a class online can have a positive impact on your face‐to‐face courses. For instance, it may improve your ability to organize and deliver content. Teaching online might have sparked your interest in the scholarship of teaching and learning, which may result in adopting new pedagogical practices. It may also improve your ability to create multiple strategies for clarification of complex concepts and to stay on schedule. Lastly, it can improve your accessibility of course materials during instructor absences and improve student learning due to the availability of repetitive course material (Scagnoli, Buki, & Johnson, 2009; Stone & Perumean‐Chaney, 2011).

How to effectively teach a quantitative class online to nonmath majors deserves a much deeper discussion than a prescription of best practices. Nevertheless, my hope is that this prescription provides a tentative playbook for colleagues to design better and more inclusive courses. In sum, this is a call to rethink the concept of *adaptation* of courses to online delivery to the *redesign* of the courses for effective teaching. Traditional teaching methods such as prerecorded lengthy PowerPoint voice‐overs are often preferred because they are familiar, easy to implement by instructors, and easy to passively follow by students (Mokhtar, Tarmizi, Ayub, & Tarmizi, 2010). However, they do little to meet desirable learning outcomes. To have effective quantitative ecology courses online, we need to break the inertia, even if it requires much initial energy. Maybe the COVID‐19 pandemic provided us with a timely incentive. It is our responsibility to provide students the quantitative resources needed to meet the challenges of the future. The stakes have never been higher.

## CONFLICT OF INTERESTS

The author has no competing interests to report.

## AUTHOR CONTRIBUTION

**Miguel Acevedo:** Conceptualization (equal); Funding acquisition (equal); Investigation (equal); Project administration (equal); Writing—original draft (equal); Writing—review & editing (equal).

## Data Availability

This paper does not include data.
